# FRET‐Based Sensor Zebrafish Reveal Muscle Cells Do Not Undergo Apoptosis in Starvation or Natural Aging‐Induced Muscle Atrophy

**DOI:** 10.1002/advs.202416811

**Published:** 2025-02-04

**Authors:** Hao Jia, Renfei Wu, Hongmei Yang, Kathy Qian Luo

**Affiliations:** ^1^ Faculty of Health Sciences University of Macau Taipa Macao SAR China; ^2^ Ministry of Education Frontiers Science Center for Precision Oncology University of Macau Taipa Macao SAR China

**Keywords:** aging, apoptosis, autophagy, FRET, live imaging, muscle atrophy, starvation

## Abstract

Muscle atrophy occurs during natural aging and under disease conditions. Muscle cell apoptosis is considered one of the main causes of muscle atrophy, while several recent studies argued that muscle cells do not die during muscle atrophy. Here, sensor zebrafish are generated to visualize muscle cell apoptosis and the engulfment of dead muscle cells by macrophages. Using these sensor zebrafish, starvation, and natural aging‐induced muscle atrophy models are established. The data showed that the diameters of muscle cells decreased in both models; however, muscle cell apoptosis is not found in the process of muscle atrophy. In starvation‐induced muscle atrophy, it also showed that the number of nuclei in muscle cells remained constant, and there is no increase in the number of macrophages in muscle tissues, both of which further confirmed that muscle cells do not die. In both models, transcriptional analysis showed that the apoptosis pathway is down‐regulated, and autophagy and protein degradation pathways are up‐regulated. All these data indicated that although there is a great reduction of muscle mass during starvation or aging‐induced muscle atrophy, muscle cells do not die by apoptosis. These findings provide new insights into muscle atrophy and can benefit the treatments for muscle atrophy‐related diseases.

## Graphical abstract

Fluorescence resonance energy transfer‐based sensor zebrafish are generated to visualize muscle cell apoptosis in live animals by color change from green to blue. Using these sensors zebrafish, starvation‐induced, or natural aging‐induced muscle atrophy models are established. The diameters of muscle cells decrease in both models; however, muscle cells do not die by apoptosis but rather remain alive by autophagy.

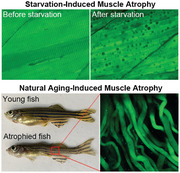

## 1 Introduction

Muscle atrophy is the loss of muscle mass and muscle strength. The major causes of muscle atrophy include aging, immobility, starvation, neurological injury, and certain diseases, such as tumors and Cushing's disease.^[^ ^1^, ^2^, ^3^ ^]^ Three main mechanisms contribute to muscle atrophy: 1) reduced protein synthesis and increased protein degradation, 2) muscle stem cell exhaustion, and 3) muscle cell death.^[^ ^4^, ^5^, ^6^, ^7^, ^8^, ^9^ ^]^

Unlike the first two mechanisms, the role of muscle cell death, especially muscle cell apoptosis, in muscle atrophy is controversial. On one side, many studies have reported that apoptosis of muscle cells occurs during muscle atrophy. In muscle tissues from aging animals, oxidative damage, mitochondria dysfunction, caspase activation, and other apoptotic markers were found to be elevated in independent studies.^[^ ^10^, ^11^, ^12^, ^13^, ^14^, ^15^, ^16^, ^17^, ^18^, ^19^, ^20^ ^]^ Apoptosis was increased in adult rabbit skeletal muscles after 2 or 6 days of immobilization, which was measured by the terminal deoxynucleotidyl transferase dUTP nick end labeling (TUNEL) assay.^[^ ^21^ ^]^ More studies showed increased levels of reactive oxygen species, caspase‐3 activity, and TUNEL signals in skeletal muscles during unloading or disuse‐induced muscle atrophy.^[^ ^22^, ^23^, ^24^, ^25^, ^26^ ^]^ Live imaging and TUNEL assays revealed that muscle cell mitochondrial damage and apoptosis contributed to denervation‐induced muscle atrophy in mice.^[^ ^27^, ^28^, ^29^, ^30^, ^31^ ^]^ Tumor cell‐derived exosomes and other factors could induce muscle atrophy through caspase‐3 activation and apoptotic cell death.^[^ ^32^, ^33^, ^34^, ^35^ ^]^ In starvation‐induced muscle atrophy, cleaved caspase‐3 was detected in muscle tissues.^[^ ^36^, ^37^ ^]^ On the other side, however, several recent studies argued that muscle cells did not undergo apoptosis during muscle atrophy induced by denervation in mouse models or induced by the normal development of intersegmental muscles in insect models.^[^ ^38^, ^39^, ^40^, ^41^ ^]^ This controversy might be partly due to the multinucleated structure of muscle cells, making it challenging to determine and characterize apoptotic muscle cells compared to single‐nucleated cells in other tissues.

In this study, we wanted to use live imaging to find out whether apoptosis is the major cause of muscle atrophy in zebrafish. To detect apoptosis in the muscle cells of live animals, we generated transgenic zebrafish Tg(*mylz2*:sensor C3) that specifically expressed a fluorescence resonance energy transfer (FRET)‐based apoptotic biosensor (sensor C3) in muscle cells. Our previous results showed that cancer and zebrafish cells expressing sensor C3 appeared green when they were alive and changed to blue when caspase‐3 was activated during apoptosis.^[^ ^42^, ^43^, ^44^, ^45^, ^46^, ^47^, ^48^, ^49^ ^]^ Here, we showed that Tg(*mylz2*:sensor C3) zebrafish enabled the detection of muscle cell apoptosis by color change at single‐cell resolution and can be used for the long‐term tracking of single muscle cell apoptosis in live zebrafish. We used starvation and natural aging to induce muscle atrophy in zebrafish and found that although there was a great reduction of muscle mass during muscle atrophy, muscle cells did not die through apoptosis.

## 2 Results

### 2.1 Generation of Transgenic Sensor Zebrafish for Visualizing Muscle Cell Apoptosis

**Figure 1:**
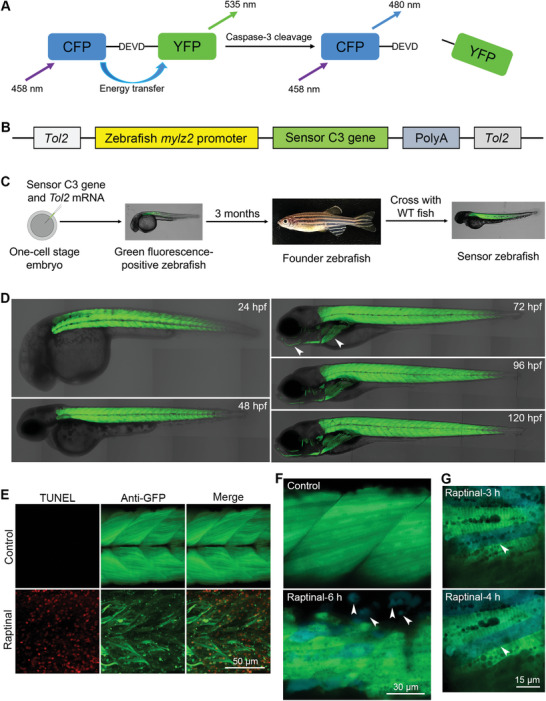
Generation of transgenic sensor zebrafish expressing an apoptotic biosensor in muscle cells. A) The structure and principle of sensor C3. B) The design and components of the *Tol2*‐based transgenic plasmid. C) The process of generating transgenic Tg(*mylz2*:sensor C3) zebrafish. D) Expression of sensor C3 in muscle cells of Tg(*mylz2*:sensor C3) zebrafish. The jaw and pectoral fin are indicated with arrowheads. The developmental time is indicated in each image. E) TUNEL assays showing the muscle cell apoptosis after the treatment of Raptinal for 8 h. F) FRET imaging showing the Raptinal‐induced muscle cell apoptosis in Tg(*mylz2*:sensor C3) zebrafish. The apoptotic bodies are indicated with arrowheads. G) Apoptotic muscle cells induced by Raptinal could maintain their morphology after caspase‐3 activation for more than 1 h. One blue muscle cell is indicated by an arrowhead to show the morphology. The size of each scale bar is indicated in each image.

To track muscle cell apoptosis in vivo, we introduced the gene of sensor C3 into the zebrafish genome to generate sensor zebrafish. The sensor C3 gene encodes a fusion protein consisting of CFP, a linker containing a caspase‐3 cleavage sequence of DEVD, and YFP (**Figure** **1**A). Because of the energy transfer from the donor CFP to the acceptor YFP, sensor C3‐expressing cells appear in green when CFP is excited using a 458 nm laser. In apoptotic cells, sensor C3 is cleaved by caspase‐3, and the FRET is abolished. As a result, apoptotic cells appear in blue when CFP is excited using the same excitation wavelength.

We cloned the sequence of a muscle cell‐specific promoter *mylz2* ^[^ ^50^ ^]^ to drive the expression of the sensor C3 gene in the *Tol2*‐based transgenic plasmid (Figure 1B). The transgenic plasmids of pSK‐mylz2‐C3 and *Tol2* mRNAs were microinjected into zebrafish eggs. After the injection, the green fluorescence‐positive zebrafish embryos were selected and cultured to the adult stage. Then, these founder zebrafish were crossed with wide‐type zebrafish to generate the transgenic sensor zebrafish Tg(*mylz2*:sensor C3) (Figure 1C). The green fluorescence of sensor C3 in the muscle cells of the trunk region of Tg(*mylz2*:sensor C3) zebrafish could be observed before 24 h post fertilization (hpf) and maintained during zebrafish development (Figure 1D). Besides the muscle cells of the trunk region, green fluorescence could also be observed in muscle cells of other regions, such as the jaw and pectoral fin, which are indicated with white arrowheads from 72 hpf (Figure 1D).

After obtaining the Tg(*mylz2*:sensor C3) zebrafish, we used a recently developed apoptosis inducer, Raptinal, which can directly damage mitochondria,^[^ ^51^ ^]^ to induce muscle cell apoptosis. Specifically, Tg(*mylz2*:sensor C3) zebrafish embryos at 24 hpf were incubated with Raptinal at a concentration of 10 µm for different time points. We first confirmed that Raptinal can induce muscle cell apoptosis using TUNEL assays. The TUNEL signals were in red, and muscle cells were labeled with anti‐GFP antibodies (Figure 1E). Then, we applied FRET imaging to detect muscle cell apoptosis in live sensor zebrafish. The sensor zebrafish were excited with a 458 nm laser. The emissions between 460 and 500 nm were collected in one channel as the CFP images, and the emissions between 520 and 550 nm were collected in another channel as the YFP images. The CFP and YFP images were merged to produce the FRET images; herein, green FRET images indicated live cells and blue FRET images indicated apoptotic cells. The results showed that after 6 h of Raptinal treatment, some muscle cells turned blue, and others already formed apoptotic bodies (Figure 1F; Figure S1, Supporting Information).

In our previous studies, we observed that within 5–10 min, blue apoptotic skin, stromal, or neuronal cells could be quickly broken down into apoptotic bodies.^[^ ^44^, ^45^, ^52^ ^]^ However, further analysis revealed that the morphology of apoptotic muscle cells could be maintained for a much longer time (at least 1 h) after caspase‐3 activation (Figure 1G). This might be due to two reasons. First, muscle cells contain many fibers that cannot be easily degraded. Second, muscle cells are merged from several myoblasts whose sizes are much bigger than the sizes of skin, stromal, or neuronal cells.

**Figure 2:**
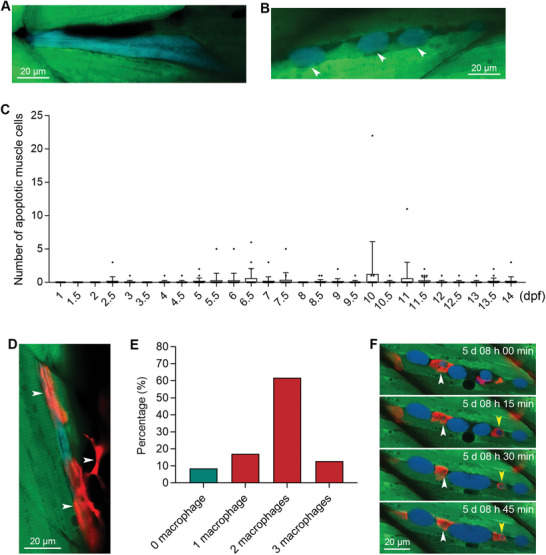
Sensor zebrafish can be used for visualizing developmental muscle cell apoptosis and the engulfment of dead muscle cells by multiple macrophages. A,B) Apoptotic muscle cells during normal zebrafish development. The blue muscle cell in was degraded into smaller fragments, indicated by arrowheads. C) The quantified results show the number of apoptotic muscle cells per zebrafish at different time points during normal development (n = 20 zebrafish for each time point). D) The engulfment of an apoptotic muscle cell by macrophages. The macrophages are indicated with white arrowheads. E) Percentages of apoptotic muscle cells that colocalized with single or multiple macrophages. F) Live tracking of the engulfment of a dead muscle cell by red macrophages. The developmental time is indicated in each image. Two apoptotic bodies engulfed by macrophages are indicated with white and yellow arrowheads, respectively. The size of each scale bar is indicated in each image.

We also examined the apoptosis of muscle cells during the normal development of zebrafish and observed some muscle cells could undergo apoptosis (**Figure** **2**A,B). We then performed a two‐week tracking of the muscle cell apoptosis during development and found that only a tiny number of muscle cells died through apoptosis (0 to 1.2 blue cells per zebrafish on average) (Figure 2C). Taken together, we showed that Tg(*mylz2*:sensor C3) zebrafish can be used to visualize apoptosis in muscle cells occurring under stress conditions and during normal development.

As sensor C3 detects caspase activation that occurs before the formation of apoptotic bodies, we were able to observe that some of the muscle cells turned blue but were still present at a large size of >100 µm (Figure 2A). To find out how these big apoptotic muscle cells are removed in zebrafish, we crossed the Tg(*mylz2*:sensor C3) zebrafish with another transgenic zebrafish, Tg(*mpeg1*:mCherry), in which the macrophages expressed red fluorescent proteins.^[^ ^53^ ^]^ Our observation revealed that red macrophages were usually colocalized with blue apoptotic muscle cells. For example, two macrophages spread out their bodies to cover one blue muscle cell, and another macrophage was approaching this apoptotic muscle cell (Figure 2D). We analyzed 47 apoptotic muscle cells during zebrafish normal development and found that 91.5% (43/47) of the blue muscle cells were colocalized with macrophages. Further analysis showed that a minority (17.0%, 8/47) of apoptotic muscle cells were colocalized with one macrophage, while the majority of apoptotic muscle cells were colocalized with two macrophages (61.7%, 29/47) or three macrophages (12.8%, 6/47), respectively (Figure 2E). These results indicated that most apoptotic muscle cells were removed by multiple macrophages.

To reveal how macrophages remove apoptotic muscle cells, we performed continued live imaging analysis. A series of images showed that two red macrophages engulfed one apoptotic muscle cell after it was broken into several smaller fragments (Figure 2F). Specifically, at 5 days, 08 h 00 min post‐fertilization, one red macrophage indicated by a white arrowhead engulfed one blue apoptotic body of the muscle cell. In the following 45 min, the three images showed that the blue apoptotic body engulfed by the red macrophage became smaller and smaller, indicating this apoptotic body was degraded within the red macrophage (Figure 2F, white arrowhead). A similar situation happened in another blue apoptotic body, indicated by a yellow arrowhead, which was engulfed and degraded by another macrophage (Figure 2F). We also observed that some un‐engulfed apoptotic bodies fused to form a larger fragment, which could increase the difficulty of engulfment by the macrophages (Figure 2F). This observation may explain why multiple macrophages are needed to remove apoptotic bodies of muscle cells collaboratively.

### 2.2 Establishment of a Starvation‐Induced Muscle Atrophy Model

**Figure 3:**
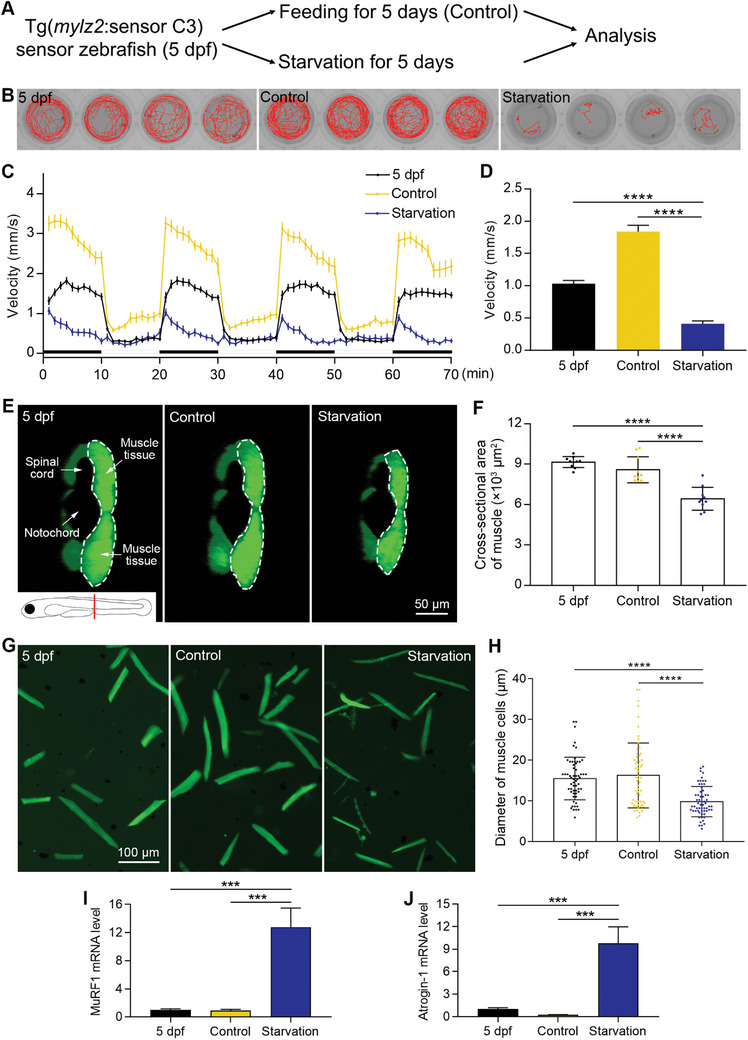
Establishment of a starvation‐induced muscle atrophy model. A) The muscle atrophy model is induced by starvation. B) The swimming trajectories of zebrafish for 5 min. C) Zebrafish locomotion tracking shows the decreased swimming velocity after starvation (n = 24 zebrafish for each group). D) The quantified results of zebrafish locomotion tracking (n = 24 zebrafish for each group). E) Images showing the cross‐sectional areas of muscle tissues. The imaging position and anatomical structures are indicated in the first image. Half of the muscle tissue is imaged, which is circled by a dashed line. F) The quantified results show that the cross‐sectional area of muscle tissues decreased after starvation (n = 10 zebrafish for each group). G) Images of dissociated muscle tissues show a decrease in the diameter of muscle cells after starvation‐induced muscle atrophy. H) The quantified results show that the diameter of muscle cells decreased after starvation (n = 60 cells for each group). I) The mRNA level of MuRF1 increased after starvation. J) The mRNA level of Atrogin‐1 increased after starvation. The size of each scale bar is indicated in each image. ^***^ *p* < 0.001, ^****^ *p* < 0.0001.

We used starvation to induce muscle atrophy in sensor zebrafish. We divided sensor zebrafish at 5 days post fertilization (dpf) into two groups: one group was fed normally for 5 days, and the other group was maintained without a food supply for 5 days (**Figure** **3**A). At the end of the experiment, a high‐speed camera was used to track zebrafish swimming trajectories (Figure 3B). We then measured the swimming velocity of the zebrafish. The results showed that the swimming velocity of the starvation group was 78% lower than that of the control group and 60% lower than that of the zebrafish at 5 dpf (Figure 3C,D).

To confirm that the condition of 5‐day starvation can indeed induce muscle atrophy, we performed 3D imaging of the muscle tissues in live sensor zebrafish and measured the cross‐sectional area of the muscle tissues. The results showed that the area in the starvation group was 25% lower than that in the control group and 30% lower than that at 5 dpf (Figure 3E,F). We then measured the diameter of each single muscle cell that was digested from muscle tissues and found that the diameter of muscle cells in the starvation group was 40% lower than that of the control group and 37% lower than that at 5 dpf (Figure 3G,H). We further measured the mRNA levels of two common muscle atrophy markers, MuRF1 and Atrogin‐1, before and after starvation treatment. The mRNA level of MuRF1 in the starvation group increased by 14.0‐fold over the control group and 12.7‐fold over the 5 dpf group (Figure 3I). The mRNA level of Atrogin‐1 in the starvation group increased by 195.6‐fold over the control group and 9.7‐fold over the 5 dpf group (Figure 3J). All these data indicated that muscle atrophy was induced in this starvation model.

### 2.3 Apoptosis Was Not Involved in the Process of Starvation‐Induced Muscle Atrophy

**Figure 4:**
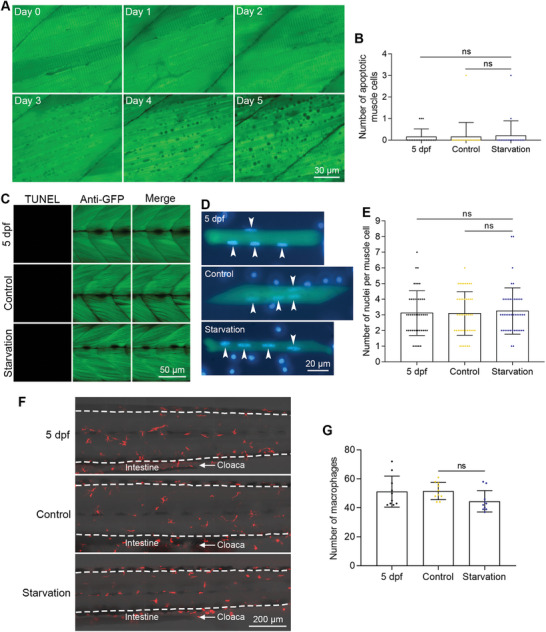
Muscle cells did not undergo apoptosis during starvation‐induced muscle atrophy. A) FRET imaging of muscle cells during zebrafish starvation. The time points after starvation are indicated in each image. B) The quantified results show the number of apoptotic muscle cells during starvation (n = 20 zebrafish for each group). C) TUNEL assays show that no muscle cells die of apoptosis after starvation. D) Nuclear staining of dissociated muscle cells. Nuclei from muscle cells are indicated with arrowheads. E) The quantified results show the number of nuclei of muscle cells after starvation‐induced muscle atrophy (n = 45 cells for each group). F) Live imaging of macrophages in the trunk regions of zebrafish after starvation‐induced atrophy. The intestine and cloaca are marked to show the images were taken from the same region. G) The quantified results show the number of macrophages after starvation‐induced muscle atrophy (n = 10 zebrafish for each group). The size of each scale bar is indicated in each image. ns: no significant difference.

To determine whether muscle cells could undergo apoptosis during starvation‐induced atrophy. We performed FRET imaging on live sensor zebrafish every day during starvation treatment. Our FRET imaging analysis showed no obvious blue apoptotic cells during the atrophy process (**Figure** **4**A,B). We also confirmed that no muscle cells died of apoptosis during starvation treatment using TUNEL assays (Figure 4C).

Muscle cells are specialized multinucleated cells in animals. If there was apoptotic cell death in muscle cells, we should be able to observe the breakdown of nuclei and the reduction of the number of nuclei. It was quite challenging to count the number of nuclei in muscle cells using DNA staining in bulk muscle tissues because of the dense structure of muscle tissues and interference from nearby stromal cell nuclei (data not shown). We digested the muscle tissue of sensor zebrafish with collagenase to obtain single muscle cells and then stained these single muscle cells with a DNA dye of Hoechst 33342. As shown here, the nuclei of muscle cells are indicated with arrowheads, and some nuclei of stromal cells are near these single muscle cells (Figure 4D). The statistical data showed that the number of nuclei in muscle cells remained constant after muscle atrophy (Figure 4E), which also indicated muscle cells did not undergo apoptotic cell death during starvation‐induced muscle atrophy.

We have shown that apoptotic muscle cells could be engulfed by macrophages (Figure 2D–F). We hypothesized that if muscle cells could undergo apoptosis after starvation treatment, the number of macrophages should increase. To test this hypothesis, we compared the number of red macrophages in muscle tissues from starved zebrafish and control zebrafish by live fluorescence microscopy. For a fair comparison, we took images of the same trunk region before and after starvation (Figure 4F). The quantified results showed that the number of macrophages did not increase after starvation treatment for 5 days (Figure 4G), indicating that there was no dramatic muscle cell death occurring during starvation‐induced muscle atrophy. All these data together indicated that the loss of muscle mass during starvation‐induced muscle atrophy was because of the reduction in the size of muscle cells rather than muscle cell apoptosis.

### 2.4 Muscle Cells Underwent Autophagy During Starvation‐Induced Muscle Atrophy

**Figure 5:**
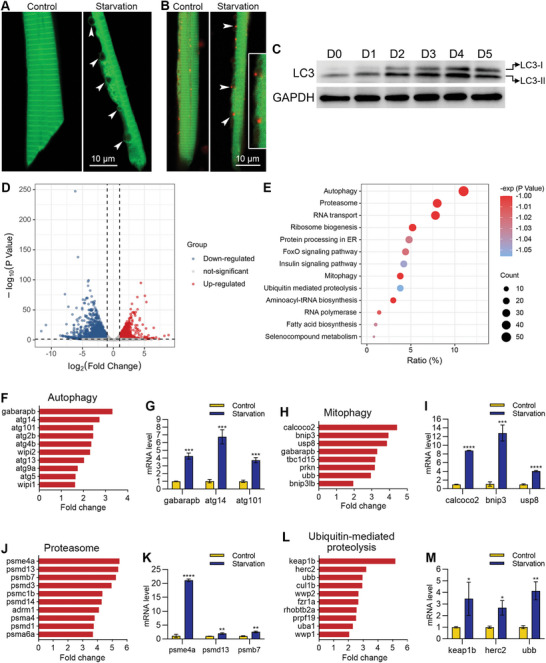
Muscle cells underwent autophagy during starvation‐induced muscle atrophy. A) High‐resolution imaging showing the unique morphology of an autophagic muscle cell. Autophagic vacuoles are indicated with arrowheads. B) Colocalization of autophagic vacuoles and lysosomes. Lysosomes are labeled with LysoTracker red, and autophagic vacuoles are indicated with arrowheads. The two autophagic vacuoles in the middle are enlarged for better illustration. C) Western blotting shows the increase of LC3‐II during starvation treatment. D) A volcano map showing the differentially expressed genes. E) The enrichment analysis shows that the autophagy pathway ranks at the top of up‐regulated genes during starvation‐induced muscle atrophy. F–M) RNA sequencing and qPCR results show the mRNA levels of up‐regulated genes in four pathways after starvation treatment. The size of each scale bar is indicated in each image. ^*^ *p* < 0.05, ^**^ *p* < 0.01, ^***^ *p* < 0.001, ^****^ *p* < 0.0001.

In the FRET images, although we did not observe blue apoptotic muscle cells, we noticed numerous small vacuoles started to appear in the muscle cells after starvation treatment for 3 days, and the size of these vacuoles became larger from 4 to 5 days (Figure 4A). Since vacuole formation is one of the hallmarks of autophagy, we wanted to investigate whether starvation treatment induced autophagy in atrophied muscle tissues. To do this, we digested muscle tissues with collagenase and performed high‐resolution single‐cell imaging. We observed many vacuoles in muscle cells from zebrafish undergoing starvation‐induced muscle atrophy and found that these vacuoles were usually at the edges of muscle cells, which is unique (**Figure** **5**A). We speculated that this unique property of muscle cells could help muscle cells maintain their ability to contract even under severe stress. We then stained the muscle cells using LysoTracker red and found the colocalization between lysosomes and these vacuoles (Figure 5B), indicating they were autophagic vacuoles. Using western blotting, we detected the increase of LC3‐II during starvation treatment (Figure 5C), which is a hallmark of autophagy.^[^ ^54^ ^]^ Interestingly, the peak of LC3‐II occurred on day 4 of starvation treatment, which may be because of the degradation of LC3‐II in autophagosomes on day 5 of starvation treatment (Figure 5C).

To get the whole picture of the mechanisms of starvation‐induced muscle atrophy, we then dissected the muscle tissues from sensor zebrafish and performed RNA sequencing analysis to compare the expression profiles between the 5 days‐starvation group and the control group. In total, there were 4337 genes differentially expressed between the starvation and control groups, including 1725 up‐regulated genes and 2612 down‐regulated genes (Figure 5D). The enrichment analysis showed that among the 13 biological processes that were significantly up‐regulated after 5 days of starvation treatment, the autophagy pathway was ranked number one (Figure 5E). The mitophagy pathway, proteasome pathway, and ubiquitin‐mediated proteolysis pathway, which are related to protein degradation, were also up‐regulated (Figure 5E). The top eight or ten genes that were significantly up‐regulated in each pathway were listed (Figure 5F,H,J,L). We performed qPCR experiments to validate the top three genes in each pathway. All the validated genes were significantly up‐regulated after starvation treatment, although the fold changes measured by qPCR experiments were not the same as RNA sequencing (Figure 5G,I,K,M).

**Figure 6:**
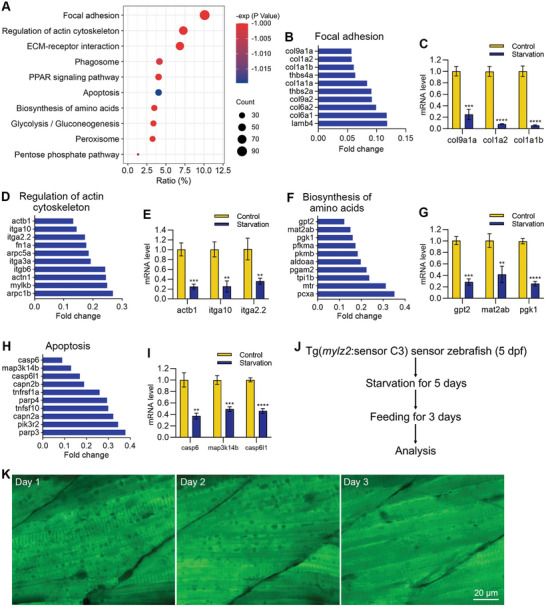
The expression of structural protein‐related genes was down‐regulated, and apoptosis‐related genes were inhibited during starvation‐induced muscle atrophy. A) The enrichment analysis shows the down‐regulated pathways during starvation‐induced muscle atrophy. B–I) RNA sequencing and qPCR results show the mRNA levels of down‐regulated genes in four pathways after starvation treatment. J) Refeeding the zebrafish after starvation treatment. K) FRET imaging shows the disappearance of autophagic vacuoles after refeeding. The size of the scale bar is shown in the image. ^**^ *p* < 0.01, ^***^ *p* < 0.001, ^****^ *p* < 0.0001.

We also analyzed the down‐regulated pathways, and the results showed that more than 10 biological pathways were significantly down‐regulated (**Figure** **6**A). The focal adhesion and regulation of actin cytoskeleton pathways were the most down‐regulated pathways, indicating starvation treatment greatly reduced the expression of structural protein‐related genes in muscle cells (Figure 6B–E). The biosynthesis of amino acids was also inhibited because of the limitation of nutrient supply during starvation (Figure 6F,G). Interestingly, the apoptosis pathway was also down‐regulated, which is consistent with our observation that apoptosis hardly occurred in muscle atrophy (Figure 6H,I).

In the next, we wanted to determine whether the autophagy in muscle cells could be rescued by food resupply. We started to refeed the zebrafish normally after the starvation treatment (Figure 6J). FRET imaging analysis showed that the autophagic vacuoles disappeared three days after the refeeding (Figure 6K).

Taken together, using these muscle‐specific sensor zebrafish, we found that zebrafish survived starvation conditions by reducing protein synthesis, and apoptosis was not involved in the process of muscle atrophy during starvation. However, muscle cells can use autophagy to maintain survival under starvation conditions.

### 2.5 No Muscle Cell Apoptosis Observed in the Process of Muscle Atrophy During Natural Aging of Sensor Zebrafish

**Figure 7:**
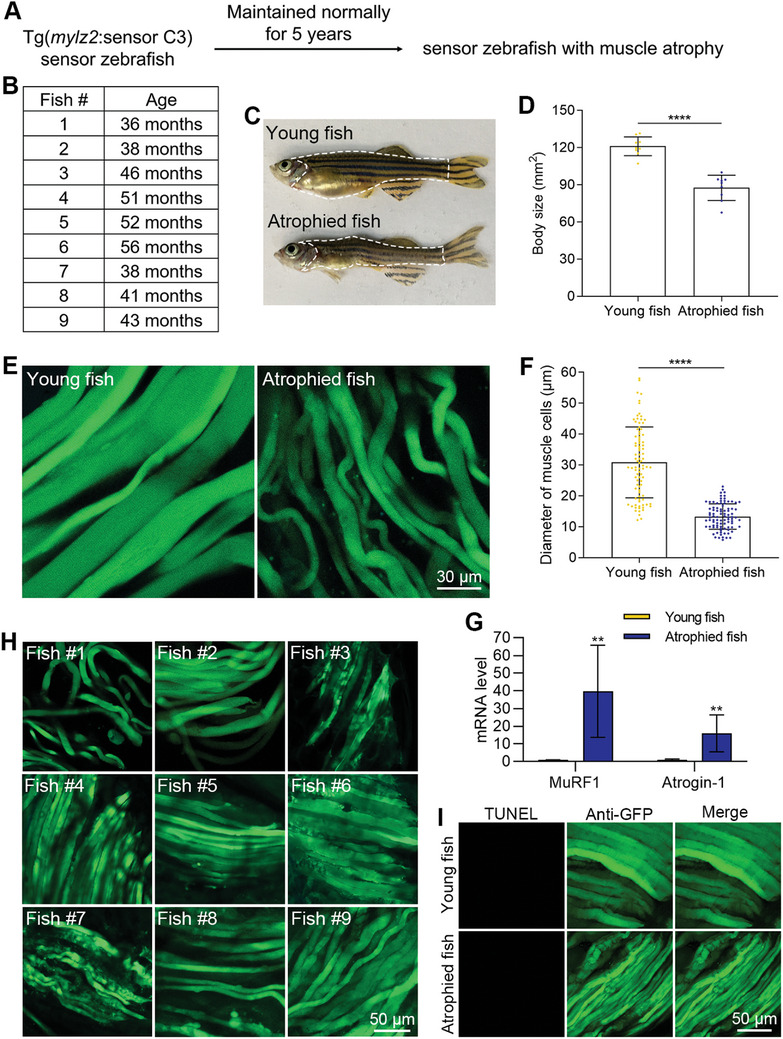
Apoptosis was not involved in the process of muscle atrophy during natural aging. A) The muscle atrophy model induced by natural aging. B) The ages of zebrafish when they were sacrificed. C) Morphologies of healthy young zebrafish and naturally aged zebrafish with muscle atrophy. D) The quantified results show the decrease in the body size of atrophied zebrafish (n = 9 zebrafish for each group). E) The decrease of the diameter of muscle cells in atrophied zebrafish. F) The quantified results show the decrease of the muscle cells in atrophied zebrafish (n = 90 cells from 9 zebrafish for each group). G) The mRNA levels of muscle atrophy markers in young and atrophied zebrafish. H) FRET imaging shows no obvious blue apoptotic signals from atrophied zebrafish. I) TUNEL assays show no muscle cell apoptosis in atrophied zebrafish. The size of each scale bar is indicated in each image. ^**^ *p* < 0.01, ^****^ *p* < 0.0001.

In addition to the starvation‐induced muscle atrophy model, using Tg(*mylz2*:sensor C3) zebrafish, we established the other muscle atrophy model induced by natural aging. We maintained Tg(*mylz2*:sensor C3) zebrafish under normal conditions for five years, which is almost the maximum longevity of zebrafish. During this long‐term tracking, some zebrafish developed muscle atrophy (**Figure** **7**A). In the first batch of 25 zebrafish, we tracked for five years and got six zebrafish displaying obvious muscle atrophy morphology. In the second batch of 25 zebrafish, until the revision of this manuscript, the tracking lasted for 44 months, and we got three zebrafish with obvious muscle atrophy morphology. The ages of these nine zebrafish when they were sacrificed are listed (Figure 7B).

To further confirm muscle atrophy in these zebrafish, first, we measured the zebrafish body size that was defined by the area of the trunk region (Figure 7C). The data showed that the body sizes of these atrophied zebrafish were significantly smaller than the young zebrafish aged from 5 to 15 months (Figure 7D). Second, individual muscle cells were obtained by treating the muscle tissues with collagenase and compared with those from young zebrafish. The results showed that the average diameter of the muscle cells in these atrophied zebrafish was significantly decreased by 57% (Figure 7E,F). We also measured the mRNA levels of two muscle atrophy markers, MuRF1 and Atrogin‐1, in young and atrophied zebrafish. The results showed that the mRNA level of MuRF1 in the atrophied zebrafish increased by 39.7‐fold over the young zebrafish, and the mRNA level of Atrogin‐1 increased by 15.1‐fold (Figure 7G).

To determine whether apoptosis occurred in the muscle cells of atrophied zebrafish, we used FRET imaging to analyze the dissected muscle cells that expressed sensor C3. The FRET images from these nine atrophied zebrafish showed that no obvious blue apoptotic muscle cells were obtained (Figure 7H). TUNEL assays were performed to confirm the FRET imaging results (Figure 7I). These data indicated that apoptosis did not occur during aging‐related muscle atrophy in sensor zebrafish.

**Figure 8:**
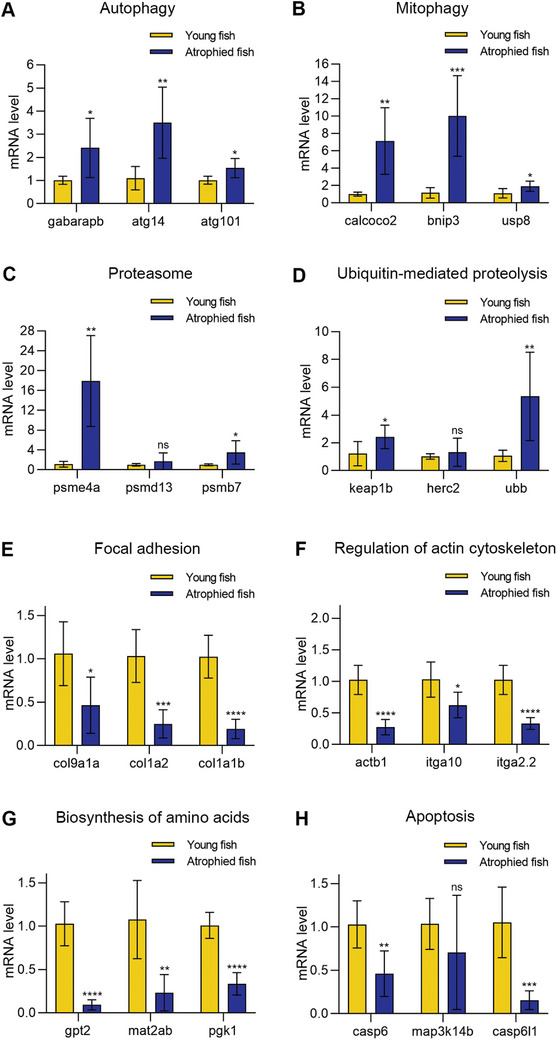
The mRNA levels of 24 genes in natural aging‐induced muscle atrophy. A–D) The genes related to autophagy, mitophagy, proteasome, and ubiquitin‐mediated proteolysis were up‐regulated. E–H) The genes related to focal adhesion, regulation of actin cytoskeleton, biosynthesis of amino acids, and apoptosis were down‐regulated. ^*^ *p* < 0.01, ^**^ *p* < 0.01, ^***^ *p* < 0.001, ^****^ *p* < 0.0001, ns: no significant difference.

Using qPCR, we measured the mRNA levels of the top genes identified in Figures 5 and 6 in the atrophied zebrafish during natural aging. Similar to starvation‐induced muscle atrophy, the autophagy and mitophagy genes were up‐regulated in the natural aging zebrafish with muscle atrophy (**Figure** **8**A,B). The proteasome and ubiquitin‐mediate proteolysis‐related genes, especially *psme4a*, and *ubb*, were also up‐regulated, indicating enhanced protein degradation in the atrophied zebrafish (Figure 8C,D). The mRNA levels of some structural proteins and genes responsible for the synthesis of amino acids were also down‐regulated (Figure 8E–G). Interestingly, the mRNA levels of apoptotic genes were down‐regulated, consistent with our finding that muscle cell apoptosis was not involved in muscle atrophy during natural aging (Figure 8H).

## 3 Discussion

Traditional animal models for muscle atrophy are mainly based on rodents.^[^ ^55^, ^56^, ^57^ ^]^ In recent years, zebrafish have become valuable model animals in biomedical studies because of their unique advantages, such as high genetic similarity to humans, ease of genetic manipulation, large sample sizes, and body transparency.^[^ ^58^, ^59^, ^60^ ^]^ In the present study, we combined sensor C3 and the transparency of the zebrafish body to generate muscle‐specific sensor zebrafish for the visualization of muscle cell apoptosis in live animals, which was quite challenging previously.^[^ ^61^, ^62^, ^63^ ^]^ Our data revealed the unique characteristics of apoptotic muscle cells and showed how the corpses of dead muscle cells were removed by phagocytic macrophages.

The traditional view over the past several decades is that during muscle atrophy, muscle mass is continuously reduced, accompanied by the loss of muscle cells through apoptosis. Based on the sensor zebrafish, two muscle atrophy models were established in this study. However, our results showed that no apparent muscle cell death was observed in either starvation or aging‐induced muscle atrophy. These findings were quite different from previous publications. Why did many previous studies conclude that cell apoptosis was involved in muscle atrophy? A recent mouse‐based study may provide a possible answer. Their results suggested that the previous TUNEL‐positive signals observed during muscle atrophy were actually from nearby stromal cells such as satellite cells, endothelial cells, fibroblasts, and macrophages. Further analysis showed that because these stromal cells and muscle cells were tightly bound together, it was not easy to distinguish the nuclei from these two types of cells in the TUNEL assay. Thus, it was very difficult to determine whether the apoptotic signals were really from muscle cells.^[^ ^39^ ^]^ A significant advantage of our transgenic sensor zebrafish Tg(*mylz2*:sensor C3) is that only muscle cells express the sensor C3 gene, which can rule out the interference of stromal cells.

Although starvation and natural aging are different methods to induce muscle atrophy, our transcriptional analysis showed that the underlying mechanisms of muscle atrophy induced by these two methods were similar. Muscle cells underwent autophagy, and apoptosis‐related genes were down‐regulated during muscle atrophy. The role of autophagy and dysregulated protein degradation in muscle atrophy was consistent with the findings in previous publications.^[^ ^36^, ^64^, ^65^, ^66^, ^67^, ^68^ ^]^ One thing that needs to be mentioned is that besides starvation and natural aging, immobility, neurological injury, and certain diseases are also common inducers of muscle atrophy. Thus, our findings require further validation in other conditions of muscle atrophy. In addition, our findings can be further validated in mammalian models and human patients.

Another interesting finding in our study is about the myonuclear domain hypothesis. According to the myonuclear domain hypothesis, each nucleus controls a defined volume of the cytoplasm of a muscle cell, and the number of nuclei adjusts to the volume of the muscle cell.^[^ ^38^, ^69^, ^70^ ^]^ Considering that the size of muscle cells decreased during muscle atrophy, the number of nuclei would also decrease. However, we found that the number of nuclei remained constant after muscle atrophy in zebrafish (Figure 4D). Recently, two independent studies also indicated that the number of nuclei in a muscle cell did not reduce after cell volume decreased. Thus, our data and these two studies provided different opinions on the myonuclear domain hypothesis.^[^ ^38^, ^39^, ^40^ ^]^

In the last several decades, many treatments for preventing muscle atrophy, such as exercise, electrical stimulation, ultrasound therapy, and nutritional therapy, have been developed.^[^ ^71^, ^72^ ^]^ However, the effects of these treatments are not satisfied. As muscle cells do not die during starvation and natural aging‐induced muscle atrophy, we thus propose that muscle masses can be recovered by approaches increasing muscle cell sizes, which could potentially benefit the treatment of starvation or natural aging‐induced muscle atrophy.

## 4 Experimental Section

### Zebrafish Husbandry

In this study, Zebrafish (AB strain) were maintained in a ZebTEC system (Tecniplast). The temperature of the zebrafish facility was 28 °C. The photoperiod of the zebrafish facility consisted of 14 h of light followed by 10 h of darkness. The adult zebrafish were fed with artemia, and the larval zebrafish were fed with paramecia or artemia. The animal experiments conducted in this study were approved by the Animal Research Ethics Committee of the University of Macau under the protocol of UMARE‐032‐2016.

### Generation of Transgenic Tg(mylz2:sensor C3) Zebrafish

The DNA sequence of zebrafish muscle promoter region of the *mylz2* gene was amplified by polymerase chain reaction (forward primer, CCGCTCGAGGATTCGCCACAGAGGAATGAGC; reverse primer, CCCAAGCTTAGTGTCCTGTACTTGAGGGGCTTAT) using wild‐type zebrafish genome as a template to drive the muscle‐specific expression of sensor C3 in zebrafish. Then, the promoter and sensor C3 gene were fused and put into a *Tol2* plasmid to generate the transgenic plasmid, pSK‐*mylz2*‐C3. The transgenic plasmids (final concentration, 50 ng µL^−1^) and *Tol2* mRNA (final concentration, 100 ng µL^−1^) were co‐injected (2–4 nL) into one‐cell stage zebrafish eggs using an MPPI‐3 microinjector (Applied Scientific Instrumentation). The green fluorescence‐positive zebrafish were selected and cultured to adulthood. These founder zebrafish were crossed with wild‐type zebrafish to generate the Tg(*mylz2*:sensor C3) zebrafish.

### FRET Imaging of Muscle Cell Apoptosis in Live Zebrafish

Tg(*mylz2*:sensor C3) zebrafish larvae were embedded in low melting point agarose gel (Promega, V2111), upon which was 0.016% tricaine solution (Sigma‐Aldrich, E10521) in an observation chamber. The sensor zebrafish were excited with a 458 nm laser using a Carl Zeiss LSM880 laser scanning confocal microscope. The emissions between 460 and 500 nm were collected as the CFP image, and the emissions between 520 and 550 nm were collected as the YFP image. The CFP and YFP images were merged to generate the FRET images that can indicate the apoptosis of muscle cells by color change. The green color indicates live muscle cells, and the blue color indicates apoptotic muscle cells.

### Induction of Apoptosis in Muscle Cells Using Raptinal

Tg(*mylz2*:sensor C3) muscle‐specific sensor zebrafish embryos at 24 hpf were dechorionated using fine forceps and then seeded into a 24‐well plate at 20 zebrafish per well. Raptinal (Merck, SML1745) was dissolved in fish water at a concentration of 10 µM and then used to treat the sensor zebrafish. After treatment, FRET imaging was applied to detect apoptosis.

### TUNEL Assay

Zebrafish larvae were sacrificed with ice water. After that, the larvae were fixed with 4% PFA overnight and then dehydrated with 30% sucrose solution for 48 h. The DNA breaks were stained using a TUNEL kit (Beyotime, C1089). The TUNEL signals were detected using a Carl Zeiss LSM880 laser scanning confocal microscope.

### Zebrafish Muscle Atrophy Models

For the starvation‐induced muscle atrophy model, Tg(*mylz2*:sensor C3) muscle‐specific sensor zebrafish larvae at 5 dpf were separated into two groups. The larvae in the control group were fed normally for 5 days, and the larvae in the starvation group were maintained without a food supply for 5 days. For the aging‐induced muscle atrophy model, adult zebrafish were maintained in the ZebTEC system with a normal food supply until the muscle atrophy phenotype occurred naturally.

### Zebrafish Behavioral Tests

The swimming ability of Tg(*mylz2*:sensor C3) zebrafish was measured using a DanioVision system (Noldus). Tg(*mylz2*:sensor C3) zebrafish were placed in a 24‐well plate (one zebrafish per well). Then, they were incubated in a water bath at 28.5 °C and under a 10 min dark and 10 min light cycle during the swimming tracking. The tracking lasted for 90 min, and the data from the first 20 min were not used for speed calculation.

### Measurement of the Cross‐Sectional Area of Muscle Tissues

Tg(*mylz2*:sensor C3) zebrafish were anesthetized and embedded in low melting point agarose gel in an observation chamber as described in the FRET imaging section. Then, z‐stack images were taken using a Carl Zeiss LSM880 laser scanning confocal microscope, and 3D construction was performed using the Carl Zeiss ZEN software. After 3D construction, the areas of the muscle tissues were calculated using Image J software. Since the depth of optical penetration of the confocal microscope cannot cover the whole trunk, half of the cross‐sectional area of the muscle tissues was measured for comparison.

### Muscle Cell Isolation from Zebrafish Larvae and Staining

Zebrafish larvae were sacrificed with ice water. Approximately 20 zebrafish were transferred into a 1.5 mL Eppendorf tube containing 245 µL of HBSS buffer and 5 µL of collagenase. Then, the tube was incubated at 37 °C with shaking for 30 min. For lysosome staining, after digestion, LysoTracker red (Invitrogen, L7528) was added into the tube at a final concentration of 50 nm and further incubated for 30 min. The isolated muscle cells were then transferred onto a slide and applied for imaging. For nuclear staining, after digestion, Hoechst 33342 (Invitrogen, H‐3570) was added to the tube at the concentration of 1 µg mL^−1^ and further incubated for 10 min. Then, the isolated muscle cells were transferred onto a slide and applied for imaging.

### Muscle Cell Isolation from Adult Zebrafish

Adult zebrafish were sacrificed with a high concentration of tricaine solution. The muscle tissues in the trunk region were dissected and transferred into a 1.5 mL Eppendorf tube containing 245 µL of HBSS buffer and 5 µL of collagenase (BioFroxx, 1904MG100). Then, the tube was incubated at 37 °C with shaking for 15 min, after which the isolated muscle cells were transferred onto a slide and applied for imaging.

### Western Blotting

Zebrafish tissues were dissected, homogenized, and then lysed using RIPA buffer supplemented with protease inhibitors. The protein concentrations of the samples were determined using Bradford assays. Around 50 µg of proteins were loaded for the sodium dodecyl sulfate‐polyacrylamide gel electrophoresis. After electrophoresis, the proteins were transferred to nitrocellulose membranes and blocked with 5% non‐fat dry milk for 1 h at room temperature. The membranes were incubated with primary antibodies (anti‐LC3, Abcam, ab48394; anti‐GAPDH, CST, 2118) at 4 °C overnight and then incubated with HRP‐conjugated secondary antibodies for 1 h at room temperature. The blots were developed with Western ECL Substrate (Bio‐Rad, 1705061) and detected using a ChemiDoc Touch imaging system (Bio‐Rad).

### RNA Sequencing and Data Analysis

At 10 dpf, Tg(*mylz2*:sensor C3) zebrafish from the control and starvation‐induced groups were sacrificed with ice water. Then, the muscle tissues of the trunk regions (≈20 zebrafish for each group) were dissected under a stereomicroscope. The dissected tissues were lysed using TRIzol (Ambion, 15596018). The samples were then sent to Novogene Company (Tianjin, China) for RNA extraction, library preparation, sequencing, and data analysis. A log2 fold change larger than 1 was set as the threshold to select the differentially expressed genes.

### qPCR Measurements

Zebrafish tissues were dissected and then lysed using TRIzol solution. RNAs were extracted and reverse‐transcribed into cDNAs using a cDNA synthesis kit (Bio‐Rad, 1708891). qPCR experiments were performed using iTaq Universal SYBR Green Supermix (Bio‐Rad, 172–5124). The fold changes of target genes were calculated using the ΔΔCT method. The primers for qPCR were listed in Table S1 (Supporting Information).

### Statistical Analyses

The data presented in this study were reported as the means ± SD, except for the data of behavioral tests, which are reported as the means ± SEM. Statistical significance was determined by *t*‐test for two‐group comparisons and one‐way ANOVA for multiple comparisons using GraphPad Prism 7 software. The significance was labeled as ^*^ *p* < 0.05, ^**^ *p* < 0.01, ^***^ *p* < 0.001, and ^****^ *p* < 0.0001, ns: no significant difference.

## Conflict of Interest

The authors declare no conflict of interest.

## Author Contributions

H.J. and K.Q.L. designed the research studies. H.J. and H.Y. performed the experiments. H.J., R.W., H.Y., and K.Q.L. analyzed the data. H.J. prepared the draft of the manuscript with input from all authors. K.Q.L. revised the manuscript.

## Supporting information



Supporting Information

## Data Availability

The data that support the findings of this study are available from the corresponding author upon reasonable request.
