# Supplementary material for: FRET‐Based Sensor Zebrafish Reveal Muscle Cells Do Not Undergo Apoptosis in Starvation or Natural Aging‐Induced Muscle Atrophy
Source: Adv Sci (Weinh). 2025 Feb 4;12(12):2416811. doi: 10.1002/advs.202416811 (PMC11947996; doi:10.1002/advs.202416811)
Supplement: Supplementary file 1 — Supporting Information [file ADVS-12-2416811-s001.docx]

**FRET-Based Sensor Zebrafish Reveal Muscle Cells Do Not Undergo Apoptosis in Starvation or Natural Aging-Induced Muscle Atrophy**

Hao Jia ^1^, Renfei Wu ^1^, Hongmei Yang ^1^ and Kathy Qian Luo ^1,2^ *

^1^ Faculty of Health Sciences, University of Macau, Taipa, Macao SAR, China

^2^ Ministry of Education Frontiers Science Center for Precision Oncology, University of Macau, Taipa, Macao SAR, China

*Correspondence: kluo@um.edu.mo (K.Q. Luo)


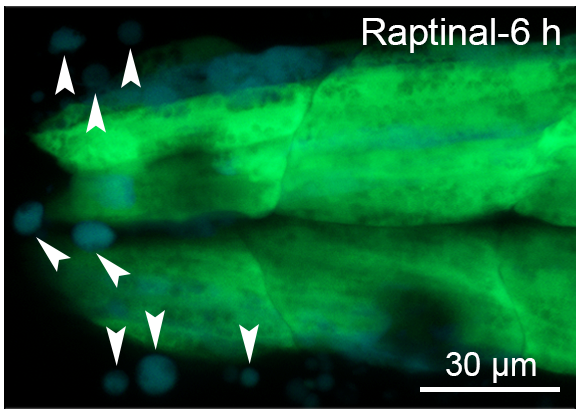


**Figure S1**. FRET imaging showing the Raptinal-induced muscle cell apoptosis in Tg(*mylz2*:sensor C3) zebrafish. The apoptotic bodies are indicated with arrowheads. The size of scale bar is indicated.

**Table S1**. Primers used in this study

| Gene name | Forward primer | Reverse primer |
| --- | --- | --- |
| actb1 | ATGGGCGTCCATGACCTTTT | ACCCTAGGGAAGTCCTGCAA |
| atg101 | TGCGCATCGACATTTCACAA | AGTGCCAATGGAGTAGGTGC |
| atg14 | CGTAGGACAGACAGGCGAAA | GAGGGACAAGCCATGACGAT |
| Atrogin-1 | GGAGCACCAAAGAGCGTCAT | CCACTCCACTCAGAGAAGGC |
| bnip3 | CTGCCACCCAAGGAGTTTCT | TAAATGTGCCTACCCGAGACC |
| calcoco2 | GGCACAACACGAACAGTGTC | AATGAACATCGCACACCCCT |
| casp6 | GGGGTGACTGGTCTGTGTTT | CGGATCCGTCGCTCTTAGTG |
| casp6l1 | CCCCAACAAATCAAACGCGA | TATCTGTGCCATTGCGGGTT |
| col1a1b | ATGACAAACGACAGCCGAGT | CTCGTTTCCCGCCCAAAAAC |
| col1a2 | TCCTAGTCGTCGCTAGATGGT | TCAGGGGGACCTGTGCAATA |
| col9a1a | TCCAGGTGCAAGGGGATCTA | GTAAATCCAACCGCACCCCT |
| gabarapb | CGTCATTCCCCCTACTTCCG | GGGTGTGTTTTCCCTTTGGC |
| GAPDH | GAGGCTTCTCACAAACGAGGA | TGGCCACGATCTCCACTTTC |
| gpt2 | GAGGCTAAGACTCTAGGAATGC | ACGTGGAAGTCTCGCAGTTT |
| herc2 | GGTGGTAGGACCGAAGAAGC | GAGAAGGCATCCTTCCCTCG |
| itga10 | TCAGACACGTTGTGGTCAAGA | TTGTCGCAAACGAGCAGTTG |
| itga2.2 | GACCTGGACCAAAAGAGCGA | CACCCACTGGAACATCTGCT |
| keap1b | TGGAGGCTTATAACGCGGATT | AGGAACACACGCAGAGGAAC |
| map3k14b | GCGAGGGACACAACATCATC | AGAGCCCGAAATCACACAGA |
| mat2ab | GTTCAGTGACTTGGTTGCTGT | TGACTGTTGCCCAATAACTCGT |
| MuRF1 | GCCCATGTGTGATGTTCACG | GTTGCTGGCGCCTAGTAGAT |
| pgk1 | AGATTGTGTGGAACGGACCC | CCTTGTCCTCAGTGTCCCAC |
| psmb7 | GAAGCGAGACCGCAGAATCA | AGTGGTTCCGGTTTTACGGG |
| psmd13 | GAACAATCTCCCGGGTGTGA | ACGTGAAAGCTCTCTCCTGC |
| psme4a | ATGGCGACCCTTGTATGAGC | TTTGAAGAAGAGCGTGGCCG |
| ubb | GTCTCCGAGGAGGCTCAGAT | GTGAGTGCATAAGCAGGGGA |
| usp8 | AGTACAACCTGTACGCCGTG | ATGGGGTTCTTGCAGTAGGC |
